# Supplementary material for: Close Temporal Relationship between Oscillating Cytosolic K+ and Growth in Root Hairs of Arabidopsis
Source: Int J Mol Sci. 2020 Aug 27;21(17):6184. doi: 10.3390/ijms21176184 (PMC7504304; doi:10.3390/ijms21176184)
Supplement: Supplementary file 1 [file ijms-21-06184-s001.pdf]

Supplementary Information

Close Temporal Relationship between Oscillating Cytosolic K^+ and Growth in Root Hairs of *Arabidopsis*

Xiangzhong Sun[1,2,#], Yuping Qiu[3,4,#], Yang Peng[3,5,#], Juewei Ning[1], Guangjie Song[1], Yanzhu Yang[1], Mengyu Deng[1], Yongfan Men[6], Xingzhong Zhao[2], Yichuan Wang[3], Hongwei Guo[3,*], Yanqing Tian[1,*]

[1] Department of Materials Science and Engineering, Southern University of Science and Technology, Shenzhen, Guangdong 518055, China
[2] School of Physics and Technology, Wuhan University, Wuhan 430072, China
[3] Institute of Plant and Food Science and Department of Biology, Southern University of Science and Technology, Shenzhen, Guangdong 518055, China
[4] Harbin Institute of Technology, Harbin, Heilongjiang 150001, China
[5] Department of Biology, Faculty of Science, Hong Kong Baptist University, Kowloon Tong 999077, Hong Kong Special Administrative Region
[6] CAS Key Laboratory of Health Informatics, Research Center of Biomedical Optics and Molecular Imaging, Institute of Biomedical and Health Engineering, Shenzhen Institutes of Advanced Technology, Chinese Academy of Sciences, Shenzhen 518055, China
[#] These authors contributed equally in this work.
[*] Correspondence: Hongwei Guo (guohw@sustech.edu.cn); Yanqing Tian (tianyq@sustech.edu.cn).

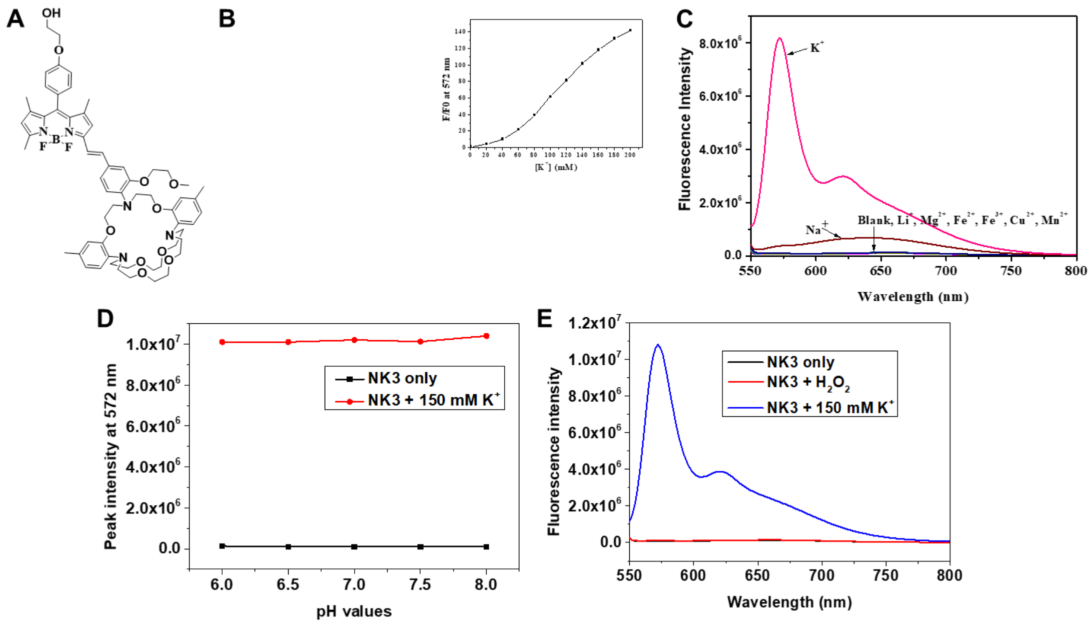

Figure S1. NK3 is a new highly selective fluorescent K^+ sensor

(A) Chemical structure of the intracellular potassium sensor NK3.

(B) Fluorescence spectra of NK3 (5.0 μM) in HEPES buffer (pH 7.4, 10 mM)/CTAB (0.5 mM) with different K^+ (0 – 200 mM). Inset is the plot of fluorescence intensity ratio F/F0 at 572 nm against K^+.

(C) Fluorescence spectra of NK3 upon addition of different metal ions. The concentrations of different metal ions are: Li^+ (10 mM), Na^+ (10 mM), Mg^{2+} (2.0 mM), Ca^{2+} (2.0 mM), Mn^{2+} (50 μM), Cu^{2+} (50 μM), Fe^{2+} (50 μM), Fe^{3+} (50 μM) and K^+ (150 mM).

(D) The plot of peak fluorescence intensity of NK3 (5.0 μM) at 572 nm against different pH values (6.0-8.0).

(E) Fluorescence spectra of NK3 (5.0 μM) upon addition of H_2O_2 (100 mM) and K^+ (150 mM).

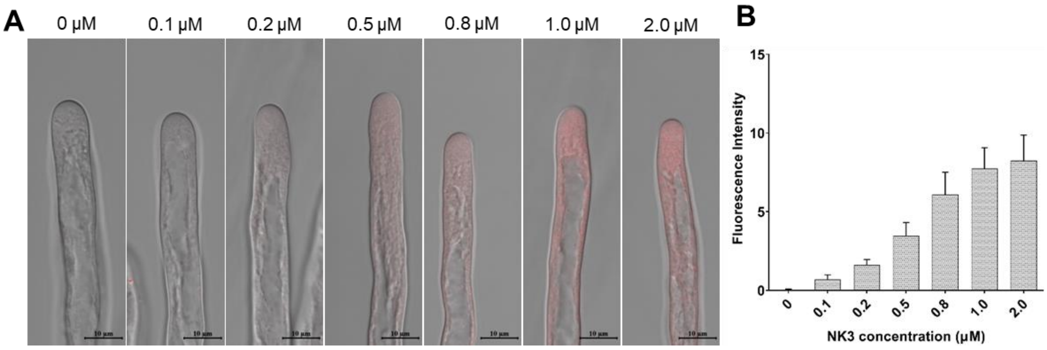

Figure S2. NK3 fluorescence intensity varies with different K^+ concentration in *Arabidopsis* root tips

(A) Root hairs of 5-day-old green seedling of Col-0 immersed in ½ MS or ½ MS medium supplemented with different concentration of NK3 (0.1, 0.2, 0.5, 0.8, 1.0 or 2.0 μM) for 6 h. Numbers indicate the concentration of NK3.

(B) Quantification of the fluorescence intensity in A. Bars represent the average intensity (mean ± s.d.) of fourteen hairs. NK3 fluorescence was measured in the approximately 30 μm^2 regions at the apex of root hairs.

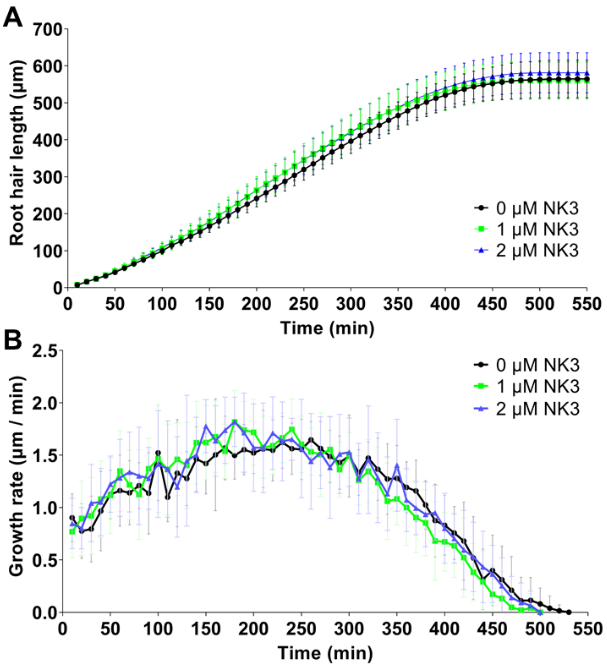

Figure S3. NK3 shows no influence on the growth of root hairs

(A) Growth curve of root hairs of 5-day-old green seedling of Col-0 transferred on medium half MS only or half MS supplemented with 1.0 or 2.0 μM NK3. Bars represent the average intensity (±s.d.) of twelve hairs.

(B) Growth rate curve of root hairs in A.

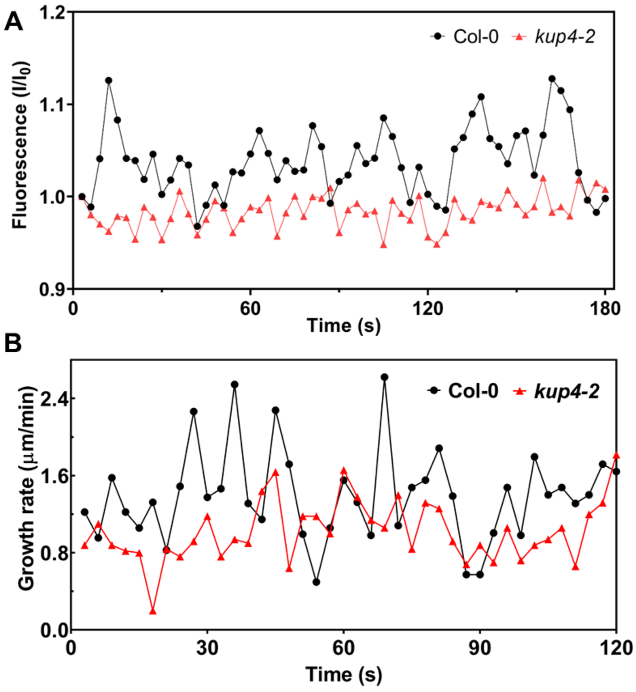

Figure S5. Cytosolic K^+ and growth oscillations are impaired in the root hairs of *kup4-2*

(A) Quantitative analysis of NK3 relative fluorescence (I/I_0) at the root hair apex of Col-0 and *kup4-2*. NK3 fluorescence was measured in the approximately 30-μm^2 regions at the hair apex. Representative results of 6 measurements are shown.

(B) Quantitative analysis of root hair growth rates of Col-0 and *kup4-2*. Representative results of 6 measurements are shown.

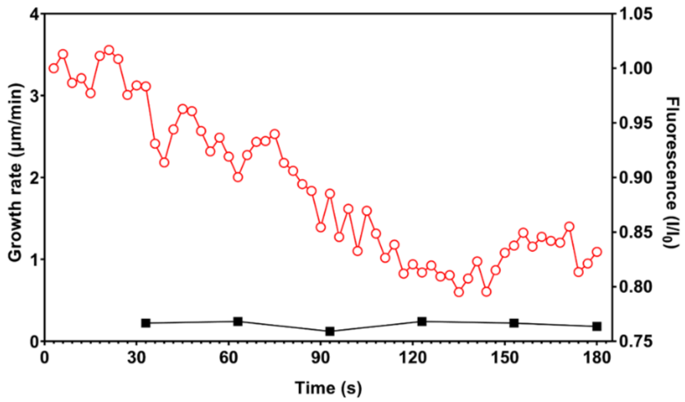

Figure S6. Maturing root hairs show a declining cytosolic K^+ level

Quantitative analysis of growth rates and NK3 relative fluorescence (I/I_0) at the root hair apex of maturing root hair. NK3 fluorescence was measured in the approximately 30-μm^2 regions indicated in A. Representative results of 6 measurements are shown.

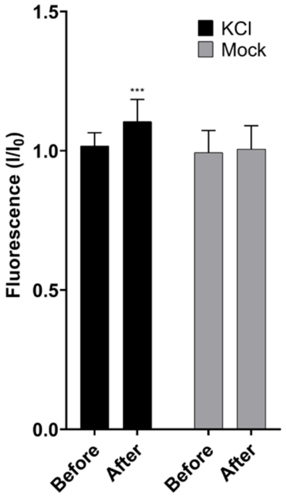

Figure S7. Effects of exogenous K^+ on the NK3 fluorescence of *Arabidopsis* root hairs Quantification of the relative fluorescence (I/I_0) of NK3-stained root hair cells treated before or after 100 mM KCl or ½ MS (Mock) treatments. Bars represent the average intensity (mean ± s.d.) of eight hairs. (Student's t-test, between seedlings before and after treated; ***P<0.001)

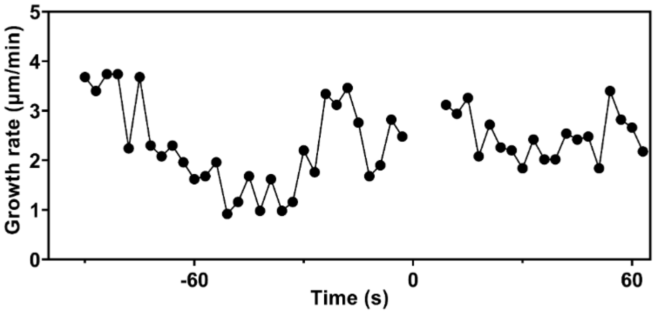

Figure S8. Effects of exogenous K^+ on the growth of *Arabidopsis* root hairs

Treatment with 100 mM KCl showed no significant influence on the growth of root hairs. Representative results of 8 measurements are shown.

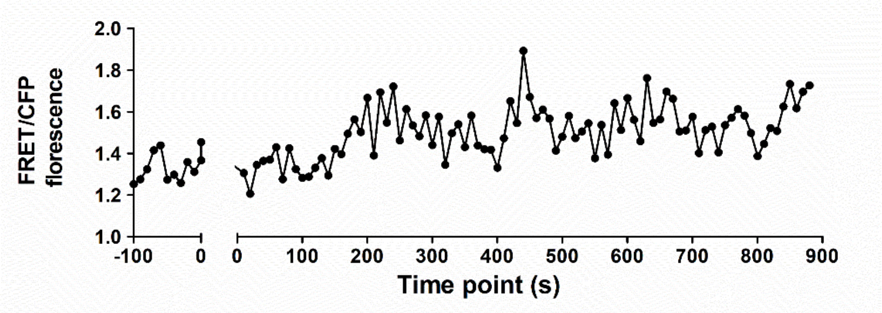

Figure S9. Effects of exogenous K^+ on the cytoplasmic Ca^{2+} level of *Arabidopsis* root hairs

Quantitative analysis of cytosolic Ca^{2+} dynamics in a growing root hair from the wild-type expressing the Ca^{2+} sensor YC3.6 were imaged every 4 s. Increase in the FRET/CFP ratio reflects an increase in cytoplasmic Ca^{2+} level. Representative results of 6 measurements are shown.

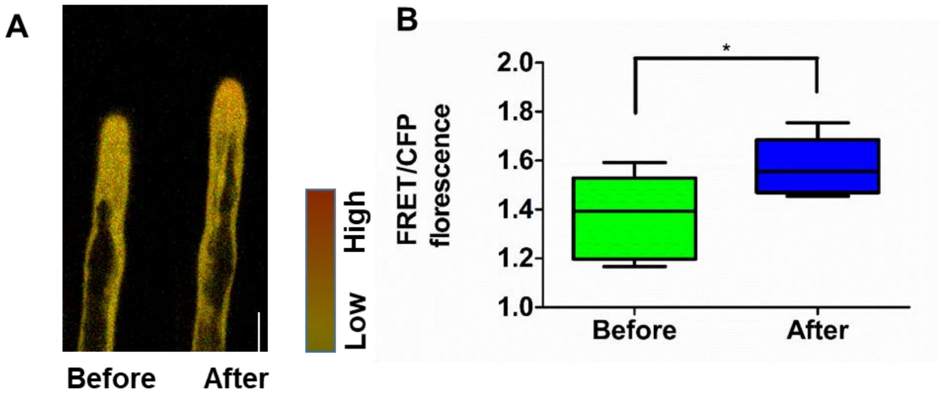

Figure S10. Effects of exogenous Na^+ on the cytoplasmic Ca^{2+} level of *Arabidopsis* root hairs

(A) 100 mM NaCl treatment induces the increase of cytoplasmic Ca^{2+} level in root hairs. Root hairs from wild-type expressing the Ca^{2+} sensor YC3.6 were imaged. Red channel represents the intensity of FRET and Green channel represents the intensity of CFP, Bar = 20 μm. Before, root hairs before treatment. After, swelling structures formed at the root hair tips within 5 minutes after treatment.

(B) Quantitative analysis of cytosolic Ca^{2+} growing root hair from the wild-type. Bars represent the average length (mean ± s.d.) of six hairs (Student's t-test, between seedlings before and after treated; *$P<0.05$).

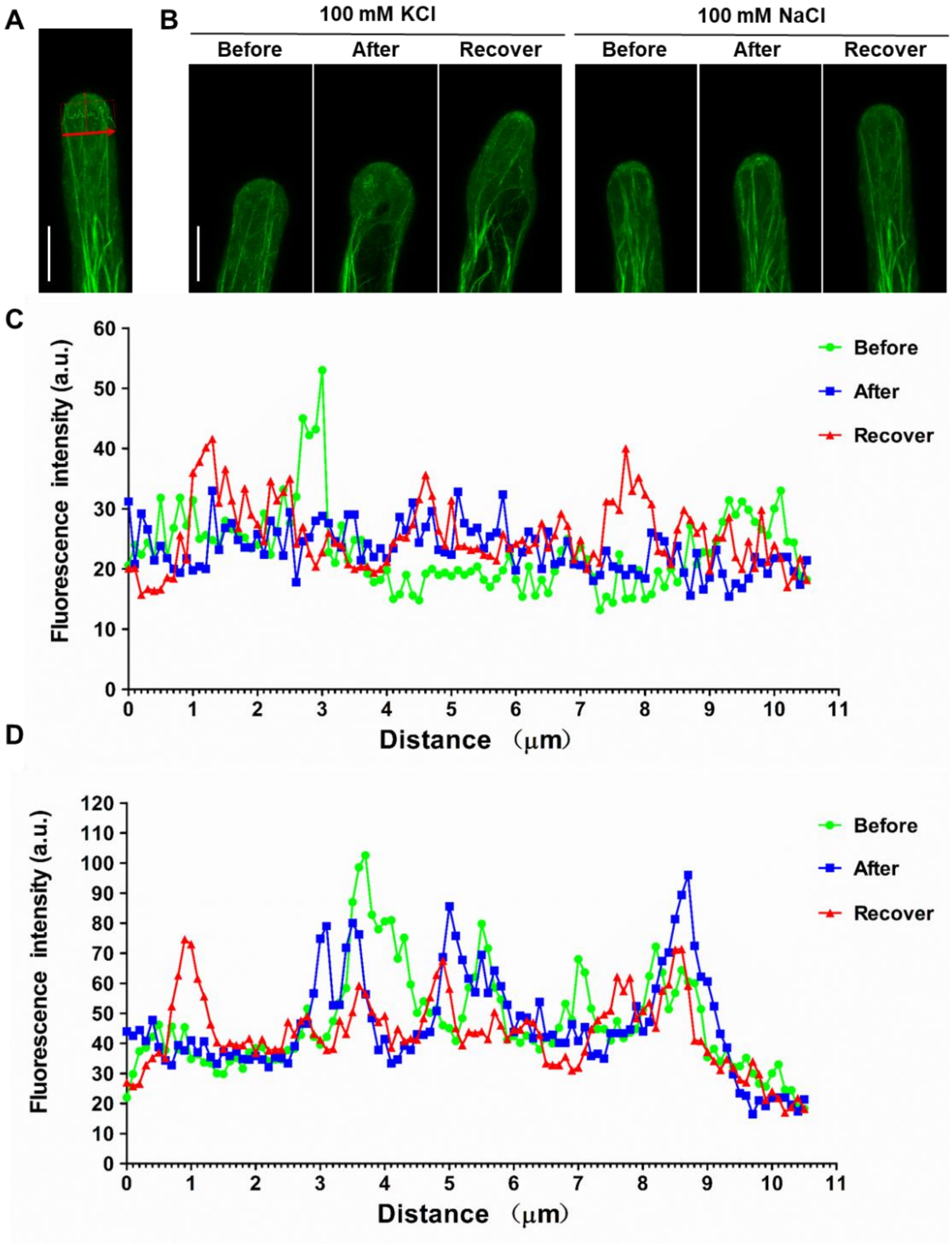

Figure S11. K^+ application influences cytoskeleton organization.

(A) The representative scheme of measurement of LifeAct-Venus in the certain position from root hair tip. Bar = 10 μm.

(B) Time-lapse Venus images of root hairs from wild-type expressing the Actin-Venus under 100 mM KCl treatment. Before, root hairs before treatment. After, swelling structures formed at the root hair tips within 5 minutes after treatment. Recover, root hairs restored to normal structure in the following 5 ~ 10 minutes. Bar = 10 μm.

(C) and (D) Quantification of the fluorescence intensity in B. (C) Under 100 mM KCl treatment and (D) Under 100 mM NaCl treatment.

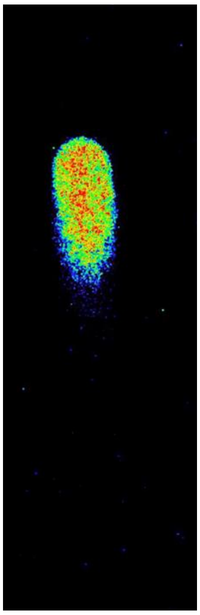

Movie S1. Cytosolic K^+ oscillations during tip growth of an Arabidopsis root hair. Images were taken every 3 s. Movie duration is 3 min. K^+ levels were pseudocolor coded according to the scale in Figure 4.
